# Extreme Weather Events in the UK and Resulting Public Health Outcomes

**DOI:** 10.3389/ijph.2025.1607904

**Published:** 2025-05-29

**Authors:** Natalie Dickinson, Llinos Haf Spencer, Shuhua Yang, Caroline Miller, Andrew Hursthouse, Mary Lynch

**Affiliations:** ^1^ Health and Life Sciences/Computing, Engineering and Physical Sciences, University of the West of Scotland, Paisley, United Kingdom; ^2^ Nursing and Midwifery, Royal College of Surgeons Ireland, Dublin, Ireland; ^3^ Faculty of Life Sciences and Education, University of South Wales, Treforest, United Kingdom

**Keywords:** public health, health impacts, health economics, UK, extreme weather events

## Abstract

**Objectives:**

Extreme Weather Events (EWEs) are increasingly frequent in the United Kingdom (UK) and can lead to adverse health outcomes, resulting in additional pressure on the NHS. The aim of this review is to investigate the health impacts of EWEs on the population in the UK, through an economic lens.

**Methods:**

A systematic review of the evidence was conducted. Seven databases were searched for studies related to the public health outcomes of EWEs.

**Results:**

48 papers met inclusion criteria: 22 flood, 25 extreme temperature, one wind. Three themes emerged: physical health impacts (predominantly temperature extremes); mental health impacts (predominantly flood-related) and socio-economic considerations (EWEs experienced disproportionately by marginalised populations).

**Conclusion:**

Whilst there is a substantial body of research on physical and mental health impacts of EWEs in the UK, there is limited evidence on socio-economic impacts, and little consideration of the economic costs. Building resilience against the health impacts of EWEs is essential. Future studies should consider incorporating cost-benefit analyses (CBA) to investigate the economic costs of EWEs on populations and health systems in the UK, and of potential mitigation efforts.

## Introduction

Extreme Weather Events (EWEs), including heatwaves, coldwaves, floods, storms and wildfires, are occurring with increased frequency worldwide, primarily driven by climate change [1]. The Intergovernmental Panel on Climate Change (IPCC) state that human-induced climate change has caused greater frequency and intensity of climate extremes globally, which has led to adverse impact on human health [2]. The impacts of EWEs in the UK are also becoming more pronounced; the UK has witnessed a notable increase in the frequency of severe weather incidents, such as heatwaves, floods and storms [3] with further changes predicted. The latest UK Climate Projections 2018 indicate a higher likelihood of warmer, wetter winters, hotter, drier summers, increased wildfires, more extreme weather events, and rising sea levels [3]. Rising temperatures, leading to extreme heat in homes and other buildings, poses an increased risk to human health, wellbeing and productivity, and has been identified as one of the priority risk areas by the UK Climate Change Risk Assessment [4].

Though a recent systematic review of EWEs across Europe exists [5], given the increase in EWEs in the UK, alongside the public health challenges of the ageing population, the healthcare crisis and socio-economic deprivation, there is a need to consider the distribution of health implications for the UK population specifically, as well as the economic cost of EWEs for the NHS.

### Aim

To investigate the impacts of EWEs on the UK population, public health and wellbeing outcomes and evaluate through a health-economic lens.

## Methods

The protocol for this systematic review was registered and published on PROSPERO following peer review [6]. The PICO [7] and eligibility criteria are presented in Table 1.

**TABLE 1:** Population, Intervention, Context/Comparator and Outcome (PICO) framework (United Kingdom, 2023).

Population	Intervention or exposure	Context/Comparator	Outcome
Population of the UK affected by EWE	EWEs (defined as heat waves, cold waves, floods, storms, wildfires and windstorms)	Routine weather events: Normal weather patterns such as average rain, sunlight, wind, air pressure	Tangible evidence relating to the impact of EWEs in the UK on public health and wellbeing outcomes

A comprehensive search strategy was developed using appropriate key words and Boolean operators to maximize the retrieval of potentially relevant studies. Key words included: (“UK” OR *alternate terms)* AND (“EWEs” OR *specific EWEs*) AND (“morbidity/mortality” OR *specific health impacts*). Full search terms can be found in Supplementary File S1. The date-range searched was November 1992 to November 2023, in line with the rise in EWEs in the past three decades [8].

Databases: Cochrane Library, CINAHL, ASSIA, PsycINFO, PubMed, Web of Science, and DARE. Grey literature, including local government reports were also included to limit publication bias.

Inclusion criteria: Papers relating to EWEs’ impact on UK population public health outcomes written in the English language from 1992 to 2023.

Exclusion criteria: Papers not related to EWEs’ influence on UK population public health and wellbeing outcomes.

**FIGURE 1:**
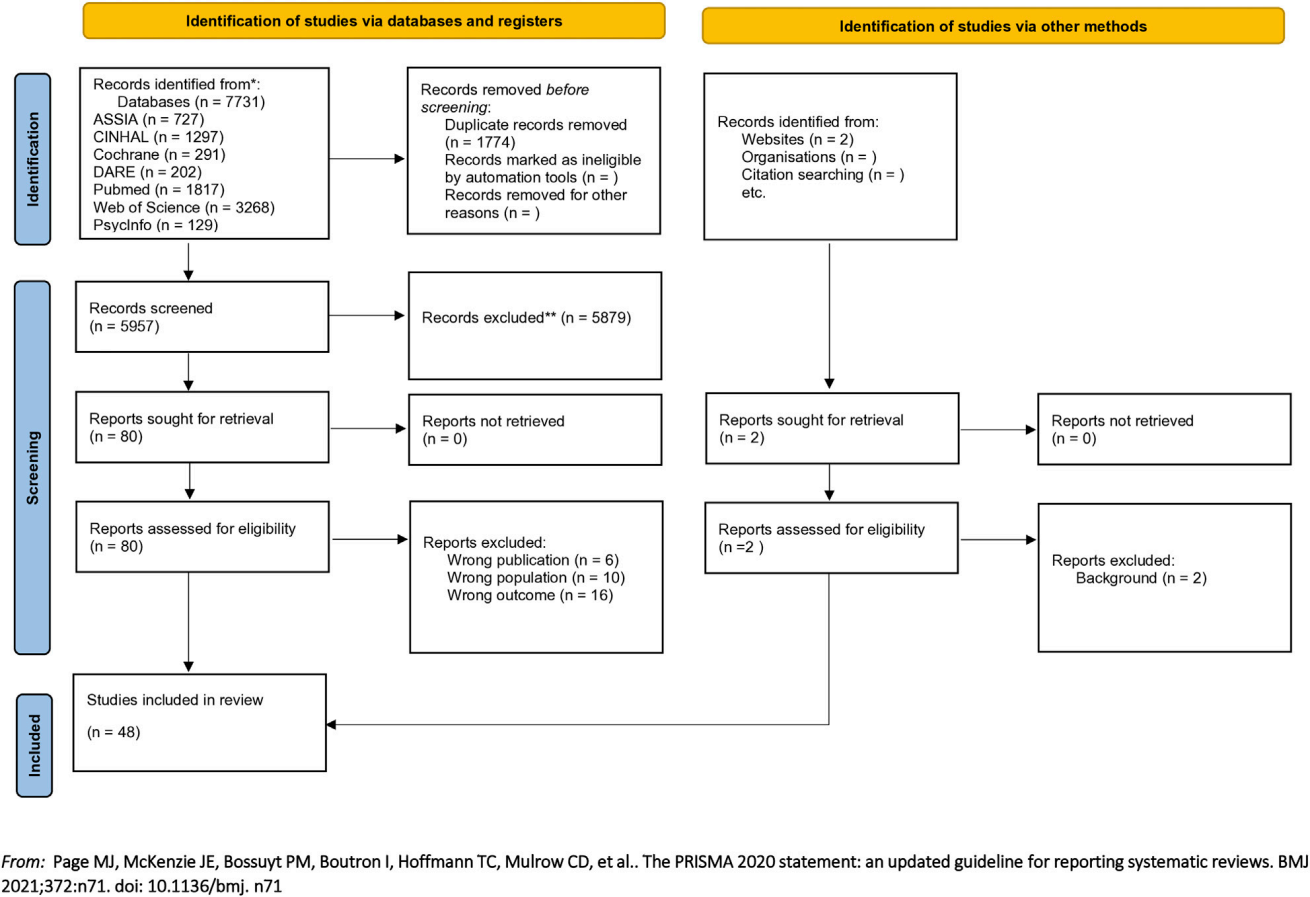
PRISMA study selection flowchart (United Kingdom, 2024).

Data management and Screening: Rayyan reference management software was used to store and manage citations [9]. Duplicates were removed in EndNOTE and Rayyan. Citations were screened on title and abstract by four members of the review team (ND, LHS, CM, and ML). Full-text articles were retrieved and further assessed for inclusion (disagreements resolved through team discussion). Figure 1 shows a PRISMA flow chart of the process [10].

Data extraction and Quality Appraisal: Two reviewers extracted data for each source. Whilst the search intentionally did not include economic terms in order to broaden the scope, the pre-defined data extraction tool included for any economic measures (see Supplementary Files S2–S4). Quality appraisal was conducted using JBI critical appraisal tools [11]. Studies were categorised as high, medium and low quality.

## Results

**FIGURE 2:**
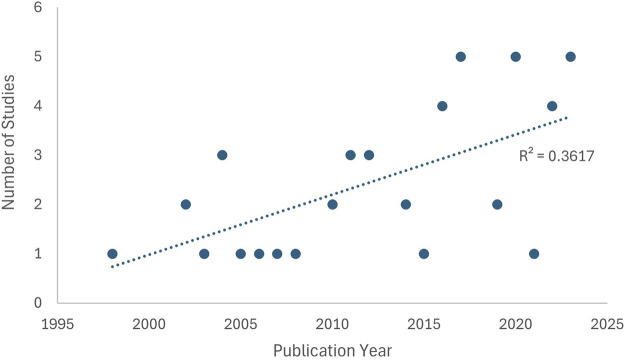
Scatter plot of included studies by publication year (United Kingdom, 1998 - 2023).

Forty-eight (48) papers met the inclusion criteria for this systematic review; 22 focussed on flood; 25 on temperature and one on wind (Table 2). Only one of the papers included a health economic measure (Disability adjusted life years, DALY’s) [12]. Figure 2 shows the included studies plotted against publication date. Though there is variability, an overall increasing trend in UK research on public health outcomes of EWEs is evident.

**TABLE 2:** Study characteristics of included extreme weather event studies (United Kingdom, 1998–2023).

#	Type of weather event	Author	Date	Country	Quality rating
1.	Flooding	Carroll et al.	2010	England	Moderate
2.	Flooding	Euripidou and Murray	2004	England	Low
3.	Flooding	Fewtrell and Kay	2008	England	High
4.	Flooding	Fewtrell et al.	2011	England	High
5.	Flooding	Findlater et al.	2023	England	High
6.	Flooding	Fothergill et al.	2021	England	Moderate
7.	Flooding	French et al.	2019	England	High
8.	Flooding	Graham et al.	2019	England	High
9.	Flooding	Hunter	2003	England and Wales	Low
10.	Flooding	Lamond et al.	2015	England	High
11.	Flooding	Mason et al.	2010	England	High
12.	Flooding	Medd et al.	2014	England	Moderate
13.	Flooding	Mehring et al.	2023	England	Moderate
14.	Flooding	Milojevic et al.	2011	England and Wales	Moderate
15.	Flooding	Milojevic et al.	2017	England	Moderate
16.	Flooding	Mulchandani et al.	2020	England	High
17.	Flooding	Munro et al.	2017	England	High
18.	Flooding	Paranjothy et al.	2011	England	High
19.	Flooding	Reacher et al.	2004	England	Moderate
20.	Flooding	Robin et al.	2020	England	High
21.	Flooding	Tapsell et al.	2002	England	High
22.	Flooding	Waite et al.	2017	England	High
23.	Heat	Alahmad et al.	2023	Worldwide including UK	High
24.	Heat	Arbuthnott and Hajat	2017	UK	Moderate
25.	Heat	Berger et al.	2023	UK	High
26.	Heat	Bryan et al.	2020	Scotland, England and Wales, UK	Moderate
27.	Heat	Cruz et al.	2020	UK	Moderate
28.	Heat	Curtis et al.	2017	England	Low
29.	Heat	Finlay et al.	2012	England	Moderate
30.	Heat	Green et al.	2016	England	High
31.	Heat	Hajat et al.	2002	England	High
32.	Heat	Heaviside et al.	2016	England	High
33.	Heat	Johnson et al.	2005	England and Wales	Moderate
34.	Heat	Kovats et al.	2004	England	High
35.	Heat	Leonardi et al.	2006	England	High
36.	Heat	Oven et al.	2012	England	Low
37.	Heat	Page et al.	2007	England and Wales	High
38.	Heat	Page et al.	2012	UK	High
39.	Heat	Rendell et al.	2020	England	High
40.	Heat	Rizmie et al.	2022	England	High
41.	Heat	Rooney et al.	1998	England and Wales	High
42.	Heat	Sahani et al.	2022	England and Scotland	High
43.	Heat	Smith et al.	2016	England	High
44.	Heat	Smith et al.	2016	England	High
45.	Heat	Thompson et al.	2022	England	High
46.	Heat	Wan et al.	2022	Scotland	High
47.	Heat	Zhang et al.	2023	UK	High
48.	Wind	Goldman et al.	2014	Organisation for Economic Co-operation and Development (OECD) countries, including UK	Moderate

Through narrative analysis, three themes emerged to describe the resulting health consequences of EWEs. These are outlined here, and discussed further in the following section.

Theme 1 Physical health impacts

Numerous studies reported a direct association between EWEs and physical health outcomes. Both heatwaves and coldwaves were linked to excess hospital admissions and increased cardiovascular and respiratory morbidity and mortality, particularly among older adults and individuals with pre-existing conditions. Several studies documented excess hospital admissions and mortality during periods of elevated temperatures. Flood events contributed to a rise in waterborne infections, skin rashes, and injuries sustained during evacuation or clean-up efforts. Additionally, storms were associated with physical injuries and power outages, impacting quality of life. One study estimates a significant cost of EWE physical impacts to the NHS.

Theme 2 Mental health impacts

Mental health consequences were prominent across the literature, with flooding, in particular, emerging as a strong predictor of psychological distress. The level of impact experienced is affected by presence of floodwater in the home, displacement and level of preparedness. Quantitative analysis of flooding’s impacts suggests mental health problems contribute more than physical impacts in terms of severity and duration. Several studies also found associations between prolonged exposure to extreme heat and increased emergency presentations for mood disorders and substance misuse.

Theme 3 Socio-economic considerations

Socio-economic status modulates both exposure to and recovery from EWE-related health impacts. Populations living in deprived areas, people with pre-existing medical conditions, females and ethnic minorities were disproportionately affected by all forms of EWE. Marginalised populations experienced delayed recovery and increased long-term vulnerability. Financial strain resulting from property damage, displacement, or income loss further contributed to negative health trajectories, exacerbated by disputes with insurance and infrastructure providers. Furthermore, the breakdown of social networks and the altered concept of ‘home’ were highlighted. Studies pointed to inequities in public health messaging, resource allocation, and emergency response, suggesting the need for more targeted, equity-focused interventions and cross-sector collaboration.

## Discussion

### Physical Health Impacts

#### Temperature Extremes

Extreme heat was more widely represented in the UK literature than extreme cold, likely due to cold temperatures being experienced over longer duration. A large body of cold-weather research exists, but as the studies do not focus on “cold waves” or “extreme cold” they were excluded from this review. Alahmad et al. investigated cold extremes, finding cardio-vascular disease (CVD) mortality to be four times greater during cold extremes than hot extremes [13]. Rizmie et al. did not observe a temperature effect on CVD, though as this study measured hospital admissions rather than mortality, the findings are not directly comparable [14]. Admissions for respiratory illness and injuries were significantly elevated during extreme cold (IRR 8.9%) and heat spells (IRR 20.9%). Furthermore, though the effect of extreme heat results in a greater number of excess admissions (6% of hospital capacity vs 3% for extreme cold days), the greater frequency of extreme cold days (0.52% vs. 0.12% of days over the 11-year study period), places a larger overall burden on the health service 911,708 vs. 97,909 days) [14].

A number of quantitative studies have demonstrated the link between excess mortality and extreme heat [13, 15–17], and between hospital admissions and extreme heat [14, 18]. Respiratory illness is the most commonly reported cause of mortality during heatwaves [15, 17, 18]. A study in Greater London found increases in emergency admissions for respiratory and renal disease related to heat, particularly among under 5s, and those over 75 (for respiratory disease) [18]. An association between heat and daily mortality was also observed during the 2003 heatwave in England and Wales, marked by a significant short-term rise in deaths. This was particularly notable in London, where mortality among those over 75 increased by 59%. Elevated levels of ozone and particulate matter were also recorded during the heatwave [16]. A study of the 1995 heatwave indicated that up to 62% (London) and 38% (rest of England and Wales) of excess deaths may be attributable to concomitant increases in air pollution [15].

The links between heat and other health outcomes are less consistent [17]. Long periods of heat cause heat strokes and contribute to an increase in other cardio-respiratory health conditions and emergency hospital admissions [13]. Alahmad et al. observed an increase in the risk for cardiovascular disease (CVD) mortality associated with heat [13]. In contrast to earlier studies, Rizmie et al. found respiratory diseases contributed less to increased admissions at temperatures >30°C (9.2%) than other conditions: metabolic diseases (25.8%) and infectious diseases (21.1%) [14].

Excess deaths were not observed in England during the 2013 heatwave, compared to similar heatwaves in 2003 and 2006 [19], whilst the 1976 heatwave [20] and the 2020 heatwaves [21] were associated with significantly higher death-rates compared to other hot periods. The reasons remain unclear, indicating the presence of unknown factors. There was no evidence to suggest the heatwaves increased the proportion of deaths occurring in different settings or that the COVID-19 pandemic altered the primary underlying causes of death during a heatwave [21].

Increased heat vulnerability has been observed in areas with high population density, significant urbanization, low coverage of green space, and elevated levels of fine particulate matter [22]. Urban heat islands (UHIs) experience higher heat-related mortality (up to 50% more) [23]. The UHI effect is compounded by the coinciding increase in air pollution. A typical heatwave in 2080 (medium-emissions scenario) could result in a mortality rate approximately three times higher than that of 2003, when accounting for factors such as population distribution changes and the UHI effect, assuming no changes in heat adaptation [23]. Geographical differences are not limited to urbanisation; in Scotland, mortality risk began to rise when temperatures exceeded 14.5°C, suggesting heat-related health impacts can occur at relatively low temperatures in cooler climates, highlighting that in places where cold weather is common, heatwaves remain an invisible threat [24]. Rizmie et al. support the notion that differing climate baselines may lead to discrepancies in effect sizes [14]. In a study of NHS 24 calls, which indicated infants and over 65s were more affected by heat, Leonardi et al. highlight that the morbidity of the elderly in relation to heat is underrepresented in hospital records, as this demographic may be less able to perceive ambient temperature, and be less inclined to seek healthcare services [25]. This suggests there may be a need for targeted support and pre-emptive intervention for older adults during heatwaves.

One study examined UV exposure, finding its health implications are complex and highly influenced by behaviours and sociodemographic factors (e.g., skin colour). Public health recommendations could be enhanced by considering both temperature and UV exposure and their effects on behaviour [26]. Drought, an effect of prolonged dry weather, can result in reduction in water quantity and quality, impacting health and wellbeing through compromised hygiene and sanitation, food security, and air quality [27]. Wildfires, an increasingly common consequence of extreme heat, can severely impact human health through inhalation of wood smoke, which impacts particularly on vulnerable populations (respiratory, cardiovascular, ophthalmic, psychiatric problems and burns [28].

#### Windstorms

One moderate quality review investigated the direct health outcomes during a powerful windstorm [29]. Direct effects occur during the storm’s impact phase, resulting in deaths and injuries from the force of the wind. Key dangers include individuals becoming airborne, being struck by flying debris or falling trees, and road traffic accidents. Furthermore, exacerbation of chronic illnesses due to limited access to medical care or medication can further affect health outcomes [29].

#### Flooding

Flooding in the UK is linked with contaminated water, leading to gastrointestinal illness supplies; Cryptosporidium was the most commonly reported pathogen in public water supply, with *Campylobacter* the commonest cause of outbreaks in private water supplies [30]. Additionally, flooding of an urban river with input from a wastewater treatment works above the flooding point was found to result in greater gastrointestinal effects than flooding caused by run-off from a more microbiologically pristine area [12]. More recent evidence suggests up to 3% of the flooded population will have a gastrointestinal illness after swallowing cumulative amounts of floodwater during the clean-up process; the use of gloves could help to reduce contact with biological contaminants, though disposable facemasks were suggested to be more effective [31]. A 2004 review highlighted three flood related chemical incidents in England following flooding, with health effects including sore throat, nausea, and stomach pains along with skin irritation. The review led to the development of a checklist for public health response and investigation for extreme flooding, which included chemical contamination identification events [32]. No recent research was found linking chemical contamination with flooding. No further recent studies on water contamination (biological or chemical) were identified, highlighting a gap in the literature.

An investigation of the long-term effects of flooding on mortality in England and Wales found an unexpected deficit of deaths in the year after flooding (post-/pre-flood ratio of 0.90, 95% CI 0.82, 1.00). No significant variation was found by age, sex, population density or deprivation, and the authors suggest displacement may be the reason for the “deficit of deaths” within the same areas pre and post flood [33].

This theme highlights the impact of EWEs on human health, with one study emphasising the limited capacity of the NHS to respond to increasing demands under the financial and operational pressures faced. Rizmie et al. estimate that excess admissions associated with extreme temperatures alone incurred an additional cost to the NHS of at least *£*20.8 million per year; they highlight that this only represents direct costs, and does not account for the broader and longer term burden to society and the health and social care system [14].

### Mental Health Impacts

#### Extreme Temperatures

Heatwaves can have positive impacts on mental health. A qualitative study on drought highlighted the positive effect of high temperatures, linked to the wellbeing effect of increased exposure to natural environments (particularly blue-spaces), though also discussed the negative impacts of prolonged extreme heat leading to drought [27]. Page et al. found that when temperatures exceeded 18°C, each 1°C rise in the average temperature was linked to a 4% increase in suicide and a 5% rise in violent suicide [34]. During the 1995 heatwave, suicide rates surged by 47%, while no significant change was observed during the 2003 heatwave, with the authors suggesting the timing of heatwaves may have relevance (those earlier in the year representing a more sudden rise in temperature) [34]. In addition, research has shown an overall increase in risk of death of 5% per 1°C increase in temperature (95% CI 2.0–7.8) in people with existing mental health conditions [35]. The greatest mortality risk was observed in younger patients and those with a diagnosis of substance misuse, with the authors noting that heatwave public health strategies should be targeted towards these groups. The mechanism for heatwaves’effect on mental health is not clear, but Page et al. suggest a combination of sociological, biological and psychological factors [34].

Whilst none of the selected studies referred to mental health impacts of cold temperatures, this is likely due to the selection criteria for this review; the effect of cold weather on mental wellbeing in the UK is extensively studied, but in relation to sustained cold temperatures rather than “extreme cold” or “coldwaves.”

#### Flooding

Mental health impact is the most significant effect of flooding [32]. This negative effect has been demonstrated quantitatively in a number of studies [12, 36–38]. Graham et al. utilised a national mental health survey to compare incidence of common mental disorder (CMD) and sociodemographic characteristics of those who had experienced flood-related damage to the home within the past 6 months, with those who had not [36]. A multiple regression model, controlling for socio-economic factors and health status, found an increase of 50% in the odds of CMD (OR 1.5, 95% CI 1.08; 2.07) in those who experienced flood damage. Fewtrel and Kay is the only study which attempted to quantify the health impacts of flooding using a standardised economic metric. DALYs were calculated based on deaths/physical injuries, infection risk, and psychological distress. DALYs ranged from 0.264 to 0.407. Mental health problems dominated the calculated health impacts. Though the method allows comparisons to be made between flood events, they may not capture all nuances such as socio-economic consequences [12].

The effect of flooding has been shown to have lasting effects. One year after flooding, prevalence of psychological morbidity in flooded participants has been observed (depression 20.1%, anxiety 28.3% and PTSD 36.2%) [39]. When compared with unaffected participants, adjusted odds ratios showed psychological morbidity to be 6-7 times higher in flooded than unaffected participants (aOR (95% CI): depression 5.91 (3.17–10.99); anxiety 6.50 (3.77–11.24); PTSD 7.19 (4.33–11.93), and 1–2 time higher in participants disrupted by the floods compared to those unaffected (aOR (95% CI): probable depression 1.56 (0.88-2.76); probable anxiety 1.61 (0.94–2.77); probable PTSD 2.06 (1.27–3.35) [39]. Similarly, it has been found that even 3 years after flooding, 7.9% of flooded respondents had probable depression, 11.7% had probable anxiety and 17.5% had probable PTSD, with higher prevalence in the flooded group compared with the unaffected group [37]. After adjustment for potential confounders, probable mental health outcomes were higher in the flooded group compared to the unaffected group, significantly for probable depression (aOR (95% CI) 8.48 (1.04–68.97) and PTSD (aOR (95% CI) 7.74 (2.24–26.79)) [39]. This is further supported by another study which used Health Related Quality of Life (HRQoL) scores [40]. Median HRQoL scores were lower in the flooded and disrupted groups compared with unaffected respondents at both two and three years post-flooding. After two years, associations between exposure to flooding and experiencing anxiety or depression were observed (aOR (95% CI) 7.7 (4.6–13.5)), persisting, though less apparent, 3 years post-flooding (aOR (95% CI) 4.3 (2.5–7.7)) [40].

The prevalence of mental health conditions appears to increase with the presence and level of flood water in the home [41]. Those with flood water above floor level had higher odds of mental health conditions (aOR (95% CI) 12.8 (9.3–17.6)) than those with water below floor level (aOR (95% CI) 3.0 (2.0–4.6)). Similarly, Waite et al. observed increased depth of floodwater to be significantly associated with all mental health conditions; depression ranged from aOR (95% CI) 4.58 (2.38–8.80) for flood depth <30 cm, to aOR (95% CI) 8.48 (4.21–17.10) for 30–100 cm, and aOR (95% CI) 14.71 (4.45–48.62) for flood depth >100 cm [39]. Paranjothy et al. also highlight subsequent disruption to services and power, more often experienced with greater flood depths, to be negatively associated with mental health outcomes [41].

Displacement from the home appears to worsen flooding’s impact on mental health. Those relocated for over six months were six times more likely to experience mental health issues than those that did not need to relocate [42]. Two further studies support this, finding displacement from home to be significantly associated with higher scores for depression, anxiety and PTSD, and individuals who had to vacate their home, along with having no previous knowledge of flooding experienced greatest levels of psychological distress [43, 44].

The prevalence of depression was higher among respondents who experienced repeated flooding compared to those affected only once, but this difference was not statistically significant after adjustment; additionally, there were no differences in anxiety or PTSD levels, and only minimal differences in overall HRQoL [45]. It could be suggested that experiencing flooding on one occasion allows those affected to build resilience for future similar events; indeed authors have observed that those without prior knowledge of flooding experienced greater distress [41]. Individuals who experienced a flood reported feeling unprepared regarding how to safely deal with flood water and potentially contaminated possessions [46]. Depression and PTSD scores were higher in individuals who were displaced and did not receive any warning, compared to those who were warned more than 12 h before the flooding. Though the difference in anxiety scores was not significant, these findings suggest that receiving advanced warning may offer protection against mental health impacts [44].

It has been observed that it is often not the flooding itself, but the recovery process that can be difficult to deal with: “project managing,” “fighting,” “loss of treasured possessions,” and “stripping-out the home” [47], underlining the value of understanding the flood recovery process to enhance resilience and preparedness for future floods. Various authors support the notion that the after-effects of flooding on mental health may be prolonged, as those affected not only need to rebuild their lives but also need to build resilience to cope with the continued threat of living in a flooding hot-spot [47–49]. Interventions that empower individuals living with ongoing flood risk are thought to potentially enhance psychological resilience [48].

It has been suggested that prioritising long-term psychological support from both formal and informal sources should be central to strategies addressing flooding’s impacts; flooding is likely to increase demand for primary care, counselling, and voluntary services, while also placing considerable strain on informal support networks [50], therefore targeting vulnerable groups is essential. Additionally, reinstating access to public transport, education, work, and health and social care services as soon as possible may be protective against mental health morbidity [39]. The evidence presented in this theme indicates substantial long-term costs to the health service, particularly in primary care, though no costings of service provision or mitigation strategies are available.

### Socio-Economic Considerations

The literature consistently shows that various socio-economic factors, such as income, age, gender, pre-existing health conditions, and family structure, are linked to increased vulnerability to flooding [41, 47, 51]. Women, ethnic minorities, those with lower income levels and older people (aged over 65 years) were found to be more affected [46]. The effects of flooding on mental health are not equally distributed; the impacts vary according to gender comorbidities, socio-economic status and level of damage or disruption experienced during an EWE. Females have been found to score more highly on PTSD, anxiety and depression scores than males, as have those in poor general health [43]. Mental health conditions were 3–5 times higher among the more socio-economically deprived Yorkshire study area than in Worcestershire, and were significantly more likely in women, unemployed, and people with comorbidities [41]. The importance of risk factors for common mental health disorders was similarly observed by Graham et al., specifically for females, those living in a deprived area, financial debt, comorbidities, and alcohol abuse [36]. Findings also suggest that lower income households are less likely to report storm or flood damage to their homes, or may be less likely to have contents insurance, resulting in under-representation in the figures generated by insurance companies [36].

Evidence highlights the inadequacy of modelling studies to measure and predict flood damage and recovery, as they cannot account for non-measurable, intangible aspects of flooding, nor for the secondary impacts encountered during the recovery process [47, 51]; both studies pointed to the meaning of home and neighbourhood, and how that can change throughout the recovery process. Higher odds of mental illness have been associated with a disruption to health and social care, and work/education amongst those living in flood-affected areas [39]. Instead of experiencing a steady progression toward normalisation, participants in a qualitative study described a pattern of highs and lows influenced by other life events, and placed importance on the quality of their interactions with the agencies involved in the recovery process [52]. The wider community is also affected, with front line workers report suffering from overwork, stress, and emotional turmoil, and that support was unavailable [52].

Geographical patterning of deprivation tends to see seaside towns housing the most deprived communities, whilst riverside locations tend to be home to the most affluent in society [36]. A mapping study revealed that areas most at risk of significant future flooding increases were coastal or situated along major estuaries, rather than those prone to river flooding [53], further compounding inequalities. The Scottish population is understudied relative to England, yet people in Scotland may be more vulnerable to temperature extremes due to the already higher mortality rate [24].

Temperature extremes are also not experienced equally. Not only has it been suggested that higher nighttime temperatures associated with greater socio-economic deprivation may contribute to excess mortality [15], research has found infant sleep to be compromised during hot weather, with the authors highlighting that even mild sleep deprivation can negatively impact concentration and learning ability [54]. Considering those from lower socio-economic groups are again likely to be the most disadvantaged, this has potential to further widen the inequality gap.

Future research should consider socio-economic conditions and segregating mortality type (by disease/age/sociodemographic factors) to calculate risk for vulnerable populations [55]. Preventative approaches to build resilience within communities are recommended [46]. The cost to the NHS of implementing such strategies could be quantified through Social Return on Investement (SROI).

### Conclusion

This systematic review highlights the pressing need for public health strategies that prioritise equity in the face of escalating EWEs. Vulnerable and marginalised populations such as older adults, people with disabilities, ethnic minorities, and those experiencing socio-economic disadvantage, are at greater risk of adverse health outcomes during and after such events. Tailored support and targeted interventions that recognise and address these intersecting vulnerabilities are critical to ensuring that no group is left behind in disaster preparedness and response.

Equally important is the development of interdisciplinary systems-thinking in preparing for future EWEs. Integrated Health and Social Care Partnerships which focus on anticipatory and preventative care [56, 57] will no doubt benefit holistic health, though collaborative efforts could be widened. Cross-sectoral collaboration, bringing together public health, social care, emergency services, urban planning, infrastructure and insurance sectors, and community organisations, is essential for enhancing system-level preparedness, improving communication pathways, and ensuring continuity of care during crises. Such joined-up efforts are also key to building long-term community resilience and mitigating the health impacts of future EWEs in the context of a changing climate.

Finally, there remains a notable gap in the application of health economics to this area. Quantifying the direct and indirect costs of EWEs to health systems, alongside evaluating the cost-effectiveness of various mitigation and adaptation strategies, is vital. Robust economic analysis will support evidence-informed policymaking and investment in sustainable, climate-resilient health systems. Addressing these three areas collectively will strengthen public health capacity to respond equitably and effectively to the increasing frequency and severity of climate-related events.

### Recommendations

• Targeted support for vulnerable groups.
• Support the integration of health and social care services to provide holistic preventative action against EWE induced health impacts.
• Develop strategies to build resilience among individuals, communities and health and care systems for future EWE’s.
• Future research exploring the impacts and mitigation strategies of EWEs should include Cost Benefit Analysis (CBA).

### Strengths

• Broad-ranging evidence synthesis of UK-specific health outcomes of EWEs from the past 30 years.
• Identified a number of gaps in the literature, significantly in the area of health economic analysis.

### Limitations

• The impacts of extreme cold are under-represented in this review This may be due to limitations of the search strategy, and “extreme cold” or “cold wave” not featuring commonly in the literature.
• The devolved nations are under-represented due to a dearth of studies in Wales, Scotland and Northern Ireland.
